# Patient-reported therapeutic benefits of herbal medicinal products in the treatment of gynecological ailments

**DOI:** 10.1186/s12906-025-04761-w

**Published:** 2025-03-11

**Authors:** Alexandra Drebka, Annika J. Scholl, Teresa Ochs, Olaf Kelber, Ralph Mösges, Esther Raskopf, Kija Shah-Hosseini, Beatrice E. Bachmeier

**Affiliations:** 1 Institute of Pharmaceutical Biology, Goethe-University, Frankfurt Am Main, Germany; 2 Phytomedicines Supply and Development Center, R&D, Bayer Consumer Health, SteigerwaldArzneimittelwerk GmbH, Darmstadt, Germany; 3 Kooperation Phytopharmaka GbR, Bonn, Germany; 4 Institute of Medical Statistics and Computational Biology, University of Cologne, Cologne, Germany; 5 ClinCompetence Cologne GmbH, Cologne, Germany

**Keywords:** Herbal Medicinal Products, Patient reported outcomes, PhytoVIS, Menstrual complaints, Menopausal complaints, Uncomplicated urinary tract infections, Pharmaco-epidemiological database

## Abstract

**Background:**

Gynecological ailments have a negative impact on quality of life and productivity. Standard treatment is associated with poor tolerability and other issues related to public health and environment. Herbal Medicinal Products (HMPs) are used traditionally for the treatment of menstrual and menopausal ailments as well as uncomplicated urinary tract infections (uUTIs) for centuries and constitute a suitable addition to current treatment options. HMPs are well tolerated, non-polluting and therapeutically efficacious as evidenced by various clinical studies. Aim of this study was to expand the evidence regarding therapeutic effectiveness of HMPs for the treatment of gynecological complaints by complementing knowledge from clinical studies with real-world evidence from patient-reported outcomes.

**Methods:**

A data set consisting of patient-reported outcomes regarding the treatment of gynecological ailments (*n* = 1658) with HMPs was taken from the pharmaco-epidemiological database PhytoVIS. After data preparation excluding all cases of herbal supplements, homeopathic preparations, or non-herbal medicinal products the remaining data (n = 1363) was grouped into the three indications menstrual complaints (*n* = 222), menopausal complaints (*n* = 301), and uUTIs (*n* = 840). We applied descriptive statistical methods (frequency and percentage) with regard to the variables “age”, “treatment duration”, “severity of symptoms”, “therapeutic benefits”, and “adverse drug reactions”. Thereafter we evaluated the therapeutic benefit of HMPs as well as adverse events.

**Results:**

The majority of the patients (82.2%) in the sample assessed the overall therapeutic effect of HMPs for the treatment of gynecological complaints as beneficial and 90.8% of them perceived no or no significant adverse events. Treatment habits differed depending on the type of complaint. In this context the majority of women with menstrual or menopausal ailments preferred to treat for time period of 1 month or longer, while those affected by uUTIs reduced the application of HMPs to the length of their symptoms. Interestingly women with even strong symptoms relied on the therapeutic benefit of HMPs.

**Conclusion:**

Real-world outcome data are an important supplement to clinical data. Our results reveal a favorable benefit-risk ratio of HMPs and help to implement them into novel therapeutic strategies to treat gynecological complaints.

## Background

Gynecological ailments can affect more than half of the world’s population and lead to considerable health issues and reduced quality of life. In particular, the high prevalence of major gynecological ailments such as menstrual and menopausal complaints and uncomplicated urinary tract infections (uUTI) has a considerable impact on productivity and health economy.

With regard to menstrual complaints, one of the biggest German health insurances (BARMER) reported in 2013 that 8.7% of women were diagnosed with "pain and other conditions associated with the female genital organs and menstrual cycle" at least once a year, affecting mainly younger women (27.9% aged 15–24 years) [1]. Regarding menopausal symptoms, around 80% of women in Germany aged between 46 and 60 suffer from menopausal complaints impacting their quality of life [2]. In 2018 about 5.5 million German women aged 40 to 79 years with menopausal ailments made use of medical services [1]. UTIs affect half of all women in Germany at least once in their lifetime [3–5], while only 12% of all men suffer from it [1]. In 2013, about 9% of all German women had a diagnosis of acute cystitis or acute urinary tract infection and most of them were aged 50 years or older. The prevalence in the age group between 20 and 29 year was about 12% per year [1].

The high prevalence of gynecological complaints leads to a negative impact on performance. About 5 – 20% of women suffering from menstrual complaints cannot carry out their daily activities due to pain related symptoms [6]. Only one out of four women is able to go to work and 80% of them are limited in their productivity. Absenteeism due to menstrual complaints is numbered with about 1.3 days per year and those who stay at home don’t disclose the real reason of illness [7]. In the United States, the economic burden due to menstrual complaints was calculated to two billion USD [8]. Preliminary data from the German Study “MenoSupport” [9] show that menopausal symptoms also have a negative impact on productivity. Accordingly, 78% suffer from physical and mental exhaustion, 66% from sleep disturbance, 46% from depressive moods and 46% from hot flashes leading in 74% of the cases to lack of concentration and in 73% to elevated perceived distress. Similar results were reported from a British study [10].

Patients with acute uncomplicated cystitis have clinical symptoms on average for 6.1 days: their activity is limited for 2.4 days, they are unable to attend classes or work for 1.2 days and are bed-ridden for 0.4 days [11].

The aim of all therapy options regarding gynecological complaints is to provide symptom relief in order to allow patients to carry out their daily activities. Standard therapies are mainly based on the application of analgetic drugs [12] or hormones [13, 14] for menstrual and menopausal complaints, or the use of antibiotics for uUTIs[15]. Cost-effective NSAIDs inhibiting cyclooxygenase-2 (COX2) and thus the production of prostaglandins are the first choice for the treatment of menstrual complaints [16], though they are associated with undesirable gastrointestinal or adverse neurological effects [12]. Another common therapy option are hormonal contraceptives [17]. However, according to German health insurance prescription counts (AOK – Allgemeine Ortskrankenkasse), the percentage of young women taking contraceptives has decreased from around 46% in 2010 to 35% in 2020. The reason for the decline is the growing awareness that contraceptives inhibit the natural hormone production [18] and can cause serious adverse events, such as venous thrombosis or an increased risk for breast cancer [19, 20].

First choice for treating menopausal symptoms is hormone therapy [21], however the WISDOM study revealed that hormone replacement therapy (HRT) can increase the risk of breast cancer or cardiovascular events [14, 22]. As a consequence, the German Society of Gynecology and Obstetrics and further international societies recommended that HRT should be used only for short-term treatment and in lower dosage [23]. In this context, many women and physicians are precarious regarding the use of HRT, which is reflected in declining prescriptions for hormone preparations [23].

Due to the complex diagnosis of UTIs, which is hard to implement for physicians in everyday practice, antibiotics are often prescribed unnecessarily [24]. However, antibiotics can damage the microbiome [11] or lead to fungal infections of the vagina [25]. Worst of all is that the use of antibiotics can increase bacterial resistance [26, 27] and pollute our food chain [28] which constitutes a major problem for our healthcare system [29].

In this context, HMPs could constitute a significant alternative treatment option solving many of these problems. For the treatment of menstrual complaints extracts of chaste tree have shown considerable efficiency in a variety of clinical studies [30] being even superior to placebo [31, 32] and potent in reducing signs of headache, irritability or breast tension [33]. The standardized extracts of black cohosh can be used for the treatment of menopausal ailments as evidenced by several clinical studies [34–36]. Recently a meta-analysis of several clinical studies revealed that cranberry products could be used, to reduce the incidence of urinary tract infections [37]. Further herbal drugs that could help to prevent recurrent urinary tract infection because of their diuretic properties are goldenrod, lovage, birch, parsley or celery [38]. Although these reports are on the first sight promising, there is still discrepancy in the results of the underlying clinical studies making it difficult to judge inasmuch HMPs can be a good alternative or supplement to standard therapies. In consequence, it is difficult to implement HMPs better into novel therapeutic strategies for the treatment of gynecological complaints.

Therefore, the aim of our study was to analyze patient-reported outcome data stored in the pharmaco-epidemiological database PhytoVIS which reflect the real world of everyday medical care. The results of our study should supplement findings from already existing clinical studies in order to scientifically evaluate the therapeutic benefits of HMPs.

## Material and methods

### Data source

A data sample taken from the pharmaco-epidemiological database PhytoVIS was used to evaluate the therapeutic benefit of HMPs in gynecological complaints. PhytoVIS was established in 2013 by the German scientific association”Kooperation Phytopharmaka “ and the Institute for Medical Statistics, Informatics and Epidemiology (now: Institute of Medical Statistics and Computational Biology, IMSB) of the University of Cologne and contains anonymized, cross-product and indication-related application data on HMPs. Data collection for the PhytoVIS study complies with the European Network of Centres for Pharmacoepidemiology and Pharmacovigilance (ENCePP) criteria on pharmacological studies. The field phase of the cross-sectional study had a duration of 3 years and was completed in December 2016 [39, 40].

The Ethics Commission of the University Hospital of Cologne positively evaluated the project for the purpose of data protection and ethical evaluation or statement (reference: 14–101). The ethical standards of the institutional and/or national research committee and with the 1964 Helsinki declaration and its later amendments or comparable ethical standards involving human participants were met. Inclusion criterion was the informed consent of the patient or the caregiver before the individual interview.

The study protocol of PhytoVIS is registered in the European Network of Centres for Pharmacoepidemiology and Pharmacovigilance (ENCePP). The clinical study number is EMA/95098/2010 (amended).

For being included into the PhytoVIS study the patients had to have experiences with the intake of herbal drugs within at least eight weeks prior to the interview and they had to give their consent to be interviewed. The defined primary endpoint was the therapeutic effect and tolerability of the products according to the user’s assessment. Source of supply and recommendation of the products were secondary endpoints of this study [39, 40].

For data collection, thoroughly trained pharmacy and medical students have conducted anonymous interviews with patients in pharmacies and general practices regarding their personal experiences with the therapeutic use of HMPs. A questionnaire designed by the “Kooperation Phytopharmaka” was used, consisting of 20 items including information on complaints/diseases, product information, drug use, concomitant factors/diseases and demographic data. Out of these questions round about 100 variables were formed. One of the variables from the complaints/disease section was the severity of complaints. In one of the questions, the participants could indicate the severity of their symptoms on an analogue scale ranging from 0 (no complaints) to 5 (strongest complaints). The treatment duration was documented in “days” or with the information "undefined longer duration". In addition, the therapeutic effectiveness could be specified with four parameter values (“very good”, “moderate-distinct”, “minimal-mild”, “unchanged or worsened”) and adverse events with four possible answers (“no adverse events”, “no significant adverse events”, “significant adverse events”, “adverse events outweigh the therapeutic effectiveness”). As one of the demographic variables, the participants could indicate their age in form of categories (e.g. "12–17 years", "18–30 years", “31–50 years”, “51–65 years”, “66–75 years”, “ > 75 years”).

### Data preparation and Data handling

The PhytoVIS database, consisting of 20,870 patients with different symptoms and diseases [40], was screened for gynecological ailments. The raw data set containing 1658 data entries was cleaned, excluding all cases regarding herbal supplements, homeopathic preparations, or non-herbal medicinal products. The remaining data (n = 1363) was grouped into the three indications menstrual complaints, menopausal complaints, and uUTIs. These data were evaluated with regard to the variables “age”, “treatment duration”, “severity of symptoms”, “therapeutic benefits”, and “adverse drug reactions”.

### Statistical analysis

Data were analyzed with descriptive statistical methods regarding frequency and percentage using IBM SPSS Statistics for Windows (version 29.0, IBM Corp. Armonk, NY, USA).

GraphPad Prism (version 10.03.275, GraphPad Software LLC, Boston, USA) was used for illustrating the results.

## Results

### Frequency of three major gynecological complaints in the sample

From a total of 1363 patients with gynecological complaints in our edited data sample the majority (62%) suffered from uUTIs (*n* = 840), followed by 22% with menopausal complaints (*n* = 301) and 16% with menstrual complaints (*n* = 222).

### Age distribution

More than half of the patients suffering from menstrual complaints (56.8%) were between 18 and 30 years old, and about one third (27.5%) between 31 and 50 years. Young adults between 12 and 17 years were less frequently affected (12.6%). Only few of the patients aged between 51–65 years (3.2%) reported about symptoms related to menstrual complaints.

Three quarters of the women with menopausal complaints (73.1%) in our sample were aged between 51 and 65 years. About a quarter of patients (23.3%) were younger (31–50 years) and only few (3.7%) were older than 66 years.

**Fig. 1:**
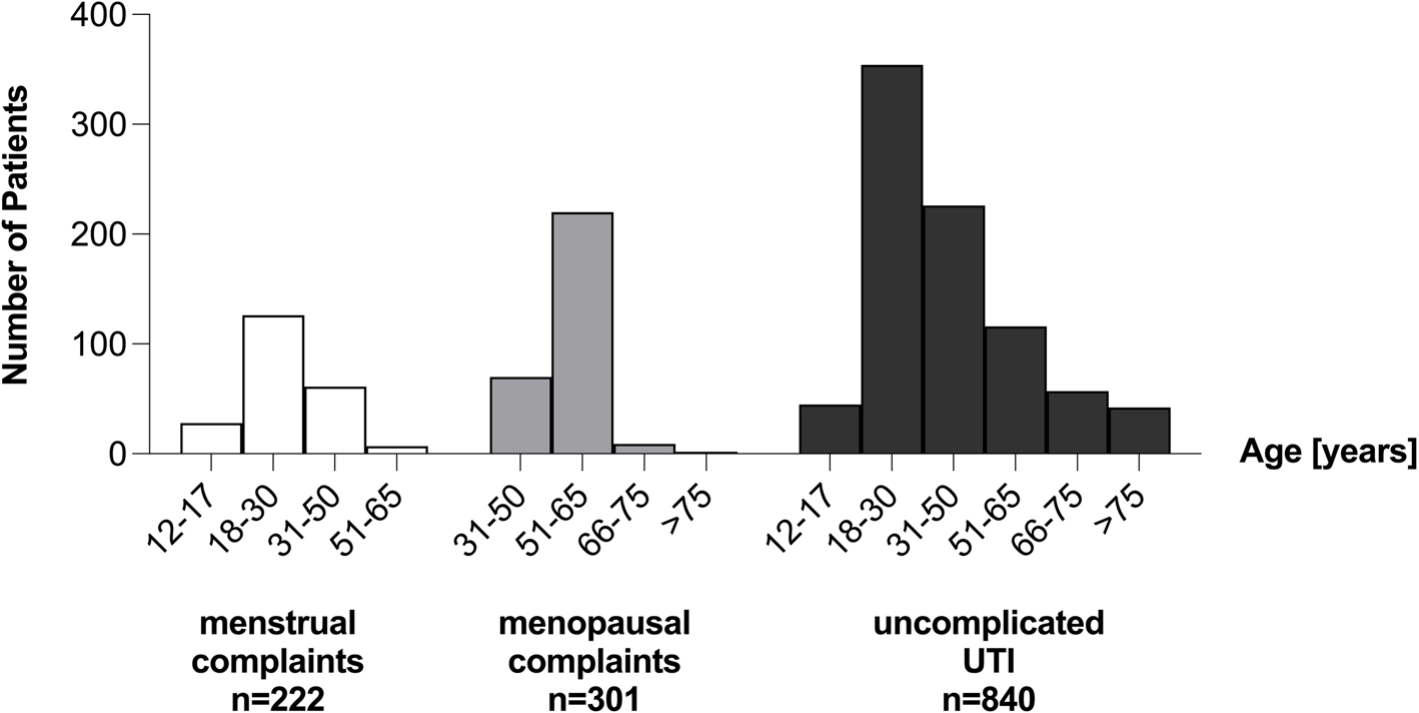
Age Distribution. In our sample 1364 patients were affected by gynecological ailments. Menstrual symptoms occurred most frequently among women aged 18–30 years, while women between 51 and 65 years suffer from menopausal complaints. Uncomplicated UTIs have been observed mainly in women aged 18–50 years

While menstrual and menopausal complaints appear only in certain age groups, UTIs affect women of all ages. Well in line this was the case also in our sample. Most frequently (42.1%) women aged 18–30 years were affected followed by more than a quarter (26.9%) who were aged between 31 and 50 years. Less than one-sixth of patients were aged between 51 and 65 years (13.8%) and only 6.8% of the women aged between 66 and 75 years. Age groups that were affected fewest were either aged under 18 years (5.4%) or over 75 years (5.0%) (Fig. 1).

### Severity of Symptoms

**Fig. 2:**
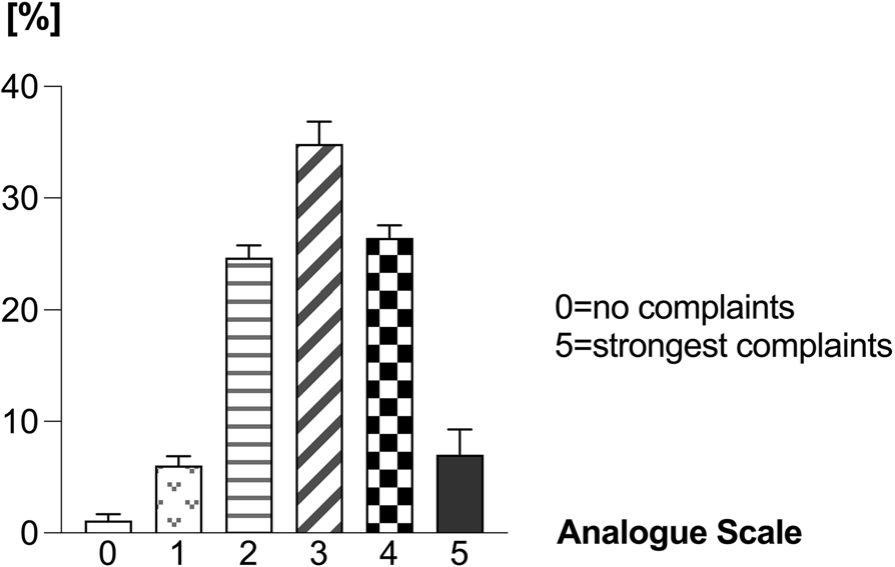
Severity of Symptoms. The majority of women with gynecological complaints using HMPs perceive their symptoms on a 6-level Likert scale between 2 and 4

The women in our sample perceived no significant differences between the three gynecological ailments regarding the severity of their symptoms. However, women suffering from uUTIsperceived their symptoms more uncomfortable than those suffering from menstrual or menopausal complaints. In this context, 9% of the women with uUTIsreported on having strongest symptoms (grade 5 on an analogue scale ranging from 0 “no complaints” to 5 “strongest complaints”) while only 4.5% of women with menstrual and 7.5% of women with menopausal ailments had similar severe complaints (Table 1, Fig. 2).

**Table 1:** Severity of Symptoms

Severity of Symptoms (6-level Likert-Scale)	Menstrual Complaints (*n* = 222)	Menopausal Complaints (*n* = 301)	uUTIs (*n* = 840)
0	0.5% (*n* = 1)	1.7% (*n* = 5)	1.1% (*n* = 9)
1	6.8% (*n* = 15)	5.1% (*n* = 15)	6.2% (*n* = 51)
2	24.5% (*n* = 54)	25.8% (*n* = 76)	23.6% (*n* = 194)
3	36.8% (*n* = 81)	34.9% (*n* = 103)	32.8% (*n* = 269)
4	26.8% (*n* = 59)	25.1% (*n* = 74)	27.3% (*n* = 224)
5	4.5% (*n* = 10)	7.5% (*n* = 22)	9.0% (*n* = 74)
no answer	*n* = 2	*n* = 6	*n* = 19

In detail, the majority of women graded their symptoms predominantly in the range of 2 (24.3%), 3 (33.9%) or 4 (26.7%) on an analogue Likert-scale reaching from 0 (no complaints) to 5 (strongest complaints). Only 6.0% of patients classified their complaints on the severity scale as grade 1. Strongest complaints were perceived by only 8.0% of the patients and only a minor share of women used HMPs without having any signs of symptoms (Table 1, Fig. 2).

### Treatment Duration

Menstrual Complaints.

Most of the patients reported that they used HMPs for an “undefined longer duration” (51.2%) or a period which was longer than one month (11.5%). About one third of them (29.5%) limited the use to the duration of their symptoms which was about 1–7 days. Only a small share of women with menstrual complaints applied HMPs for a time period of 8–30 days (7.8%).

Menopausal Complaints.

The vast majority of women suffering from symptoms used HMPs for a “longer undefined period” (79%) or longer than one month (11.9%). Only a small share of women reported that they used HMPs between 1–30 days (9.1%).

Uncomplicated UTIs.

**Fig. 3:**
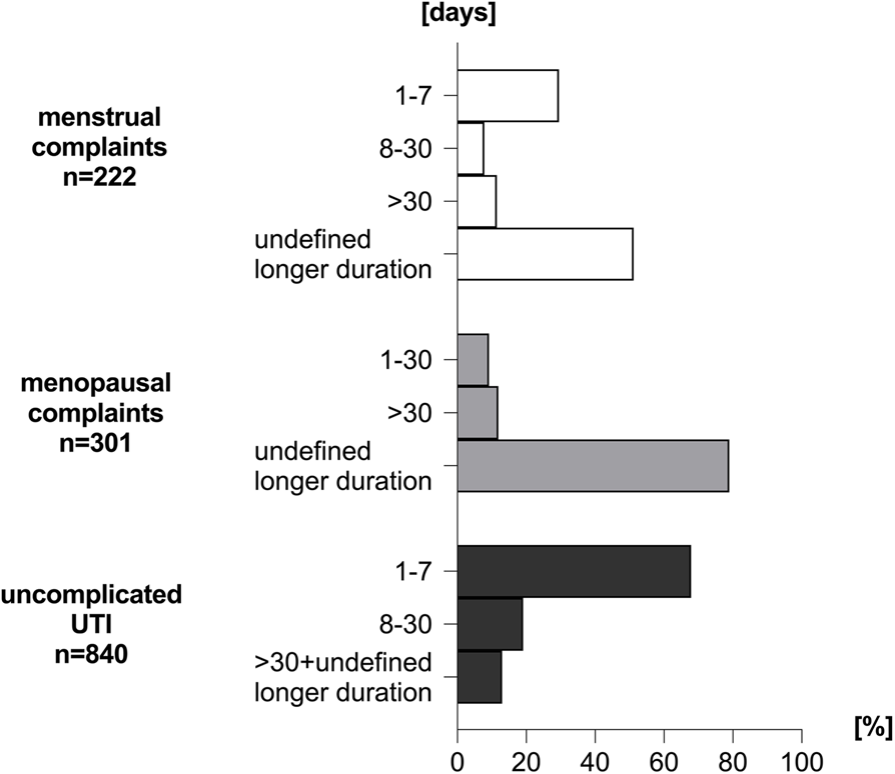
Treatment Duration. In our sample HMPs were used by most of the women for an undefined longer duration to treat menstrual and menopausal complaints. For symptoms of uUTIs, HMPs have been used mainly for a shorter time period

It appears that women applied HMPs only when they had symptoms. In this context two third of the women (67.9%) did not exceed a treatment duration of 7 days. The remaining third used HMPs for a time period of 8–30 days (19.1%) and only few treated longer than 30 days or for a longer undefinable period (13.0%) (Fig. 3). This is in line with the recommendations for the application of HMPs for the treatment of uUTIs.

As a considerable share of women with menstrual complaints (29.5%) took HMPs for only 1 – 7 days (although the recommendation is to apply the medication for 3 months) we wished to know how those women experienced the therapeutic effectiveness. Remarkably, women who took the HMPs for only up to 8 days experienced a comparable therapeutic effectiveness to those taking the medication for 30 days or longer. In this context the majority of women taking the medication for a short time period (87.5%) perceived the therapeutic effectiveness as “very good” or “moderate to distinct” and likewise did the majority of women who applied HMPs for a timer period of at least 30 days (or longer) (82.6%) (Table 2).

**Table 2:** Association between Treatment Duration and Therapeutic Effectiveness for Menstrual Complaints

Treatment Duration	Therapeutic Effectiveness			
	**very good**	**moderate-distinct**	**minimal-mild**	**unchanged or worsened**
1–7 days (*n* = 64)	37.5% (*n* = 24)	50% (*n* = 32)	10.9% (*n* = 7)	1.6% (*n* = 1)
> 30 days or undefined longer duration (*n* = 136)	29.4% (*n* = 40)	53% (*n* = 73)	12.5% (*n* = 17)	4.4% (*n* = 6)

For menopausal complaints or uUTIs these considerations are obsolete due to the low share of women who did not take the HMPs as recommended.

Due to these unexpected results, we analyzed if the women with menstrual complaints perceived the timepoint of onset of the therapeutic effectiveness differently depending on the treatment duration (short versus long). Interestingly, women who applied HMPs only for a short time period perceived the onset of effectiveness earlier than those taking the medication for a time period over 30 days. In detail of those women preferring a short treatment time about two third recognized the effectiveness already after few minutes (17.2%) or few hours (45.3%), a little less than one third after 1 day (to up to one week) and only a minor share thereafter or could not say when it began. More than one third of the women who applied the medication for more than 30 days could not tell anymore when they perceived the onset of therapeutic effectiveness and a little less than half of them reported that it took at least one week until they felt that HMPs were efficacious. Only a minority (in sum about 10%) felt that HMPs have an immediate effect within minutes or hours (all results summarized in Table 3).

**Table 3:** Association between Treatment Duration and Onset of Therapeutic Effectiveness for Menstrual Complaints

Onset of Therapeutic Effectiveness	Treatment duration	
	**1–7 days (*n* = 64)**	**30 days or longer (*n* = 136)**
after few minutes	17.2% (*n* = 11)	2.9% (*n* = 4)
after few hours	45.3% (*n* = 29)	8.1% (*n* = 11)
> 1 day	26.6% (*n* = 17)	5.1% (*n* = 7)
> 1 week	1.6% (*n* = 1)	42.6% (*n* = 58)
cannot say	6.3% (*n* = 4)	39.7% (*n* = 54)
no answer	3.1% (*n* = 2)	1.5% (*n* = 2)

### Therapeutic benefits of Herbal Medicinal Products

Taking all three gynecological ailments together, more than 80% of the patients in the sample evaluated the overall therapeutic effect of HMPs for the treatment of gynecological complaints as beneficial.

More specifically the therapeutic effectiveness was predominantly perceived as “very good” (39.4%) or “moderate-distinct” (42.9%). About one tenth (13.2%) of the patients perceived a “minimal to mild” effectiveness and a minority (4.6%) reported that HMPs were not efficacious or worsened the symptoms.

Menstrual Complaints.

About half of the women (50.9%) described the effectiveness as “moderate to distinct” and more than one-third (31.5%) reported a “very good” relief of symptoms. About one tenth of the patients (12.6%) described the effectiveness of HMPs as “minimal to mild”. Only few patients (5.0%) did not benefit having unchanged or worsened symptoms.

Menopausal Complaints.

The majority of women perceived the treatment with HMPs as “very good” (33.9%) or “moderate to distinct” (45.5%). A “minimal to mild” effect of HMPs was perceived by 15% and again, only few (5.6%) women did not benefit from HMPs.

Uncomplicated UTIs.

**Fig. 4:**
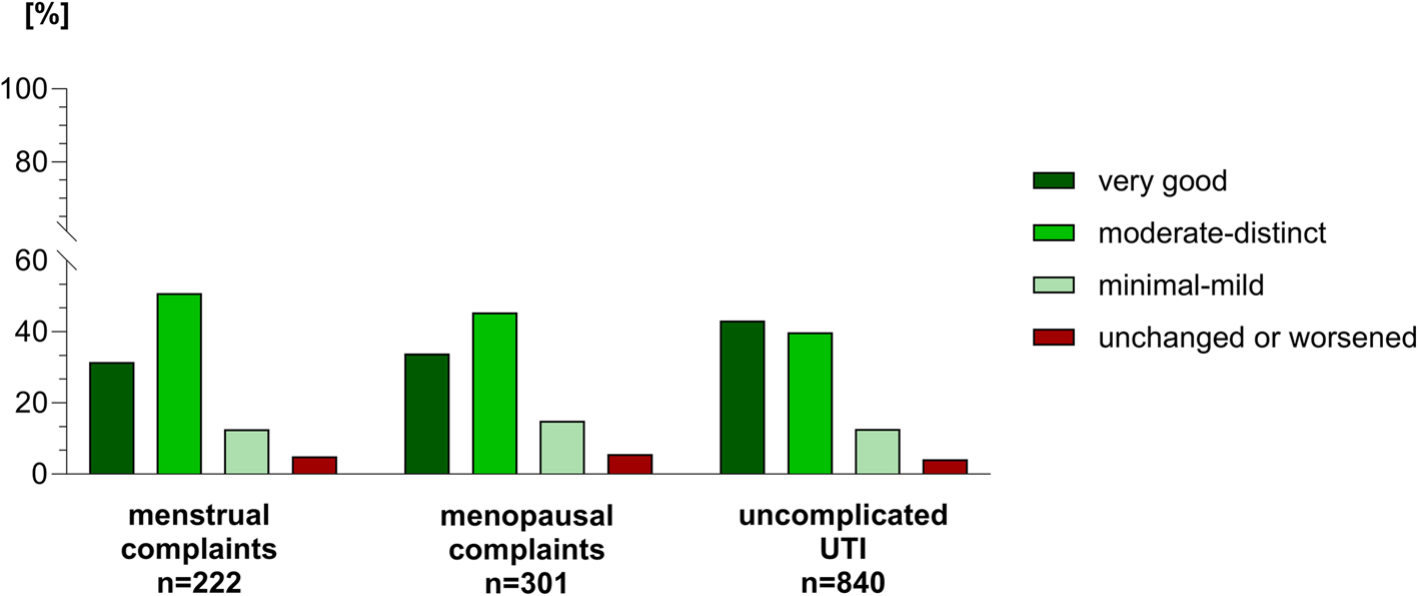
Therapeutic Effectiveness of HMPs. Patients in our sample perceived the therapeutic benefits of HMPs for the treatment of gynecological complaints predominantly as “very good” or “moderate to distinct”

Noteworthy, most women in the sample described that they experienced the therapeutic effectiveness of HMPs as “very good” (43.2%) or “moderate to distinct” (39.9%) for the treatment of uncomplicated cystitis. Comparable to menstrual and menopausal symptoms, the percentage of women reporting on “minimal to mild” (12.7%) or unchanged/worsened symptoms (4.2%) was low. All results are illustrated in Fig. 4.

### Analysis of Adverse Drug Reactions of Herbal Medicinal Products

**Fig. 5:**
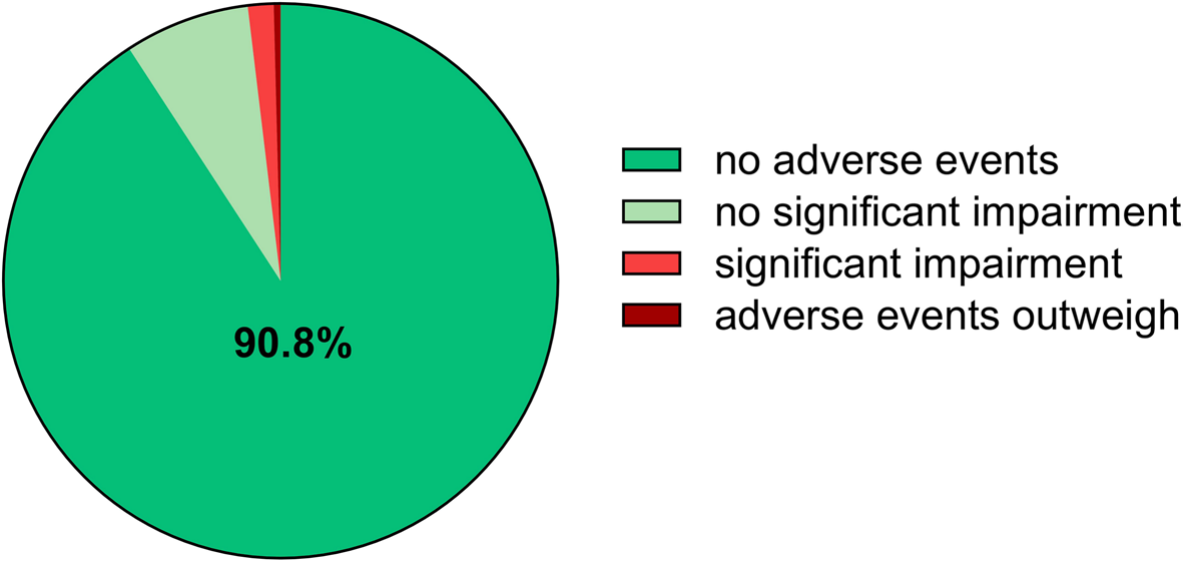
Adverse Drug Reactions. Most patients in our sample did not perceive significant adverse drug reactions when they applied HMPs to treat their gynecological complaints

The incidence of adverse drug reactions of HMPs for the treatment of gynecological complaints was very low in the analyzed sample. Overall, the majority of patients perceived “no adverse events” (90.8%) or “no significant adverse events” (7.3%). Only a small percentage of patients reported that they had “significant adverse events” (1.5%) or “adverse events that outweigh the therapeutic effectiveness” (0.4%) (Fig. 5). When opposing the percentage of patients who did not experience any adverse events or had only minor adverse events (in sum 98.1%) to those who experienced adverse events (1.9%) and taking into account that the percentage of patients evaluating the overall therapeutic benefit of HMPs for the treatment of gynecological complaints as very positive (80%), the benefit-risk of HMPs can be evaluated as excellent.

## Discussion

The demand for novel treatment strategies based on HMPs is currently rising among patients [41, 42] in particular in the field of gynecological complaints yet the acceptance and the willingness of physicians to recommend HMPs is still minor. The reason for this could be that the number of randomized clinical trials investigating the effectiveness of HMPs for the treatment of gynecological complaints is limited. In addition, the few clinical trials performed so far often fail to provide a convincing proof of effectiveness because the results between the studies are not consistent or sometimes even contradictory. The reasons for this could be a low number of participants, heterogenous patient samples, missing laboratory or other measurables parameters that could indicate the effectiveness of HMPs, or the drug extract preparations used as study medication are of uncertain origin [43].

In this context real-world evidence (RWE) is an essential addition to clinical studies, and patient-reported outcomes (PROs) are valuable measurement parameter to delineate the therapeutic benefit of HMPs.

The pharmaco-epidemiological database PhytoVIS is to the best of our knowledge the only registry based on patient-reported outcomes (PROs) regarding the therapeutic effectiveness of HMPs and provides real-world evidence of everyday medical care.

Our results from analyzing a sample taken from PhytoVIS contributes significantly to gain knowledge on the therapeutic benefits and the applicability of HMPs for the treatment of gynecological complaints.

In our sample HMPs have been used by younger and older women likewise to treat gynecological complaints. This is in line with the growing popularity of HMPs and the increasing sales in pharmacies and prescriptions in Germany since 2020 [44].

Age distribution for gynecological ailments in the analyzed sample matches well with the characteristics of the basic population: The majority of women with menstrual complaints in our data set were aged 18–30 years (56.8%) and 31–50 years (27.5%) which adds up to 84.3%.This is in line with an Irish study that determined a prevalence of 91.5% within a age range from 18 to 45 years [45].

Regarding menopausal complaints, most of the women in the analyzed data set were aged between 51–65 years, which is well in accordance with information provided by the RKI (Robert-Koch Institute, Germany), revealing that menopausal symptoms are the most common reason for women over 50 years of age to visit a gynecologist [46].

In the case of uUTIs, the frequencies in the predefined age groups of our analyzed data set does not match perfectly with the frequencies in similar age groups reported by the German health insurance BARMER in the year 2013. While in our analyzed sample women between 18 and 30 years and between 31 and 50 years were affected more often than other age groups, the majority of women affected in the German population is 66 years or older [1]. The differences could be due to the fact that our analyzed sample consists of only patients who use HMPs to treat their ailments. Moreover, our data was collected by a voluntary survey conducted in pharmacies and not by a statuary health insurance. This leads us to the assumptions that (i) younger woman could be more open to alternative treatment options and (ii) patients who go to pharmacies and participate in surveys are predominantly younger that 66 years of age. Although there is a discrepancy in age distribution between our sample and the basic German population concerning uUTIs, we are confident, that this does not have a significant influence on the evaluation of the therapeutic effectiveness.

Regardless of age and kind of ailment most of the women in the analyzed sample felt quite impaired by their symptoms and in this context, two third of them reported that the severity of their symptoms ranged between 3 and 4 on a 6-level Likert Scale. Likewise, it is remarkable that women with even strong complaints relied on the therapeutic effectiveness of HMPs. The severity of the symptoms is a good indicator to evaluate inasmuch women are affected in their daily activities and their quality of life. Therefore, we assume that the working women in our data sample with gynecological complaints were less productive, which can have a significant impact on economy [7–9].

Although the therapeutic benefits of a reasonable number of HMPs are traditionally conveyed and acknowledged by the HMPC (Committee on Herbal Medicinal Products) of the EMA (European Medicines Agency), the scientific proof of evidence of their effectiveness for the treatment of gynecological complaints has not yet been delivered. Therefore, we analyzed the sample regarding therapeutic effects perceived by the patients with gynecological complaints who took HMPs to treat their symptoms.

The patients in our sample perceived the therapeutic effectiveness predominantly as “very good” or “moderate to distinct”. Only a minor share of all women did not perceive any therapeutic effectiveness of HMPs. At the same time, the vast majority of women did not experience any adverse events or had only minor adverse effects. In this context, the benefit-risk ratio can be evaluated as favorable.

This is well in line with a series of randomized controlled clinical studies that rated the therapeutic effectiveness of HMPs in comparison to placebo or a standard medication as very good or at least non-inferior. In this context pharmaceutical preparations of chaste tree were perceived as efficacious and even non-inferior to placebo or fluoxetine by women with premenstrual syndrome [47, 48]. Likewise, results from clinical studies revealed that women with menopausal complaints and their attending gynecologists rated the therapeutic benefits of black cohosh in comparison to hormone replacement therapy as significantly better with less adverse reactions like vaginal bleeding, breast and abdominal pain and leukorrhea [49] or at least as non-inferior [36]. A standardized combination extract made from rosemary, centaury and lovage tested in a randomized controlled study was found to be non-inferior to antibiotics in the treatment of uUTI [50]. However, a recently published review points to the risk that non-antibiotic strategies for the treatment of UTIs results in poorer clinical outcomes leading to subsequent antibiotic treatment and pyelonephritis [51]. Therefore, caution is advised and although the patients in our sample perceived the therapeutic benefits of HMPs for the treatment of UTIs as efficacious at the timepoint of the survey we could not analyze any possible complications thereafter.

One of the reasons why patients chose HMPs, is their good tolerability and the minor occurrence of adverse drug reactions. Well in line, about only 2% of the patients in our sample perceived significant adverse effects of HMPs or effects that outweigh the benefits. Because 80% of patients reported that their symptoms were noticeably alleviated, a favorable benefit-risk ratio can be attributed to HMPs.

HMPs are predominantly used for self-medication and patients tend to seek fast and easily accessible information in the internet. Only few ask physicians for consultation on HMPs, because patients often feel embarrassed or as not taken seriously if they ask for alternative treatment options [52]. Patients would rather prefer to ask pharmacists, however physicians as well as pharmacists are limited in their time to consult in depth because they do not get adequately reimbursed for it. Therefore, patients often do not know how to intake HMPs properly, which has an impact on treatment effectiveness, as well as on adverse drug reactions or interactions with other drugs.

The most popular HMPs for the treatment of menstrual and menopausal complaints are made of standardized extracts from *Vitex agnus-castus* L*.,* fructus [30] or *Cimicifuga racemosa* (L.) Nutt., rhizome [34, 35]. The HMPC of the EMA recommends in its final assessment reports on *Vitex agnus-castus* L*.,* fructus a treatment duration of 3 months [53], and for *Cimicifuga racemosa* (L.) Nutt., rhizome a maximum treatment duration of 6 months [54], however, to the best of our knowledge, no studies have been performed so far analyzing if shorter treatment durations could have a similar therapeutic effect.

In this context, we analyzed if the patients in our sample took the HMPs “properly”.

The habits of taking their medication allows to speculate on the degree of patient literacy regarding HMPs. The majority of women in our sample treated their menopausal and menstrual complaints for a “longer undefined period” and thereby demonstrated that they are well informed about the fact that they should treat not only during the couple of days when they have symptoms. Interestingly, one quarter of women with menstrual complaints took their herbal medication only when they had symptoms and even more remarkable was that these women perceived the therapeutic effectiveness of HMPs as good as did the women who treated for a time period of 30 days or longer. Moreover, the majority of women who treated for a shorter period of time recognized the onset of therapeutic effectiveness early within the first couple of minutes and hours after application of the medication, while most of the women who treated for a longer period of time realized the effectiveness of HMPs only after at least one week. It is also notable that about 40% of women taking HMPs to treat their menstrual complaints could not determine the exact time point of onset of therapeutic effect. For menopausal complaints and uUTIs, an analysis of similar correlations was obsolete as the majority of women took the medication as recommended.

Our study of real-word outcome data taken from the pharmaco-epidemiological data base PhytoVIS to assess the therapeutic effectiveness and safety of HMPs for the treatment of gynecological complaints is the first of its kind and provides novel and essential insights which can complement the results from clinical studies.

As other data collections PhytoVIS has strengths and limitations. A big advantage is the large patient cohort, which makes statistical analysis possible and meaningful. Additionally, the collective of patients/participants has not been preselected and thereby is diverse and representative. The application of advanced data collection techniques guarantees a decrease in the frequency of systematic and random measurement errors.

One of the limitations of the data set is that the age of the patients was categorized into predefined age groups. This affects the statistical analyses regarding the group size and the frequencies. Another limitation was that the treatment duration was protocolled in the two categories "days" and "longer undefined period”: in particular the category “longer undefined period” leaves the question unanswered what exactly the patient perceives as a longer undefined period (one week, one month, one year etc.).

The severity of the complaints was surveyed on a six-level analogue scale (Likert-Scale), with only the endpoints defined as “no complaints” and “strongest complaints”. Thus, the middle answer categories offer free interpretation for the patient.

The results from our study reveal that HMPs are an attractive treatment option for women who seek an effective alternative to hormone preparations, NSAIDs or the recurrent use of antibiotics.

Especially in view of complementary or alternative therapy options for gynecological complaints, satisfying therapeutic effectiveness paired with limited adverse events resulting in a favorable benefit-risk ratio is particularly important, in order to implement HMPs in novel treatment strategies.

## Conclusion

Our analysis of patient reported outcome data from the real world of everyday medical practice reveals that HMPs are a beneficial treatment option for menstrual and menopausal complaints as well as uUTIs. The here presented results are an important supplementation to the sometimes-contradictory data already published from the few clinical studies on the effectiveness of HMPs for the treatment of gynecological complaints.

Representative surveys in the German population have recently shown that the demand for HMPs [41, 42], especially among women and in particular the younger generation, is increasing. Gynecological ailments are suitable for the use of HMPs because the courses of disease are not life-threatening.

However, it is crucial to conduct additional research to determine which herbal drugs are best suited for which ailment and how safe and efficacious the treatment with HMPs is in particular in combination with other medication. In addition, treatment durations for HMPs should be reconsidered as shorter periods could be equal sufficient lowering the risk for possible side-effects or interactions with co-medication.

The combined analysis of real-world Evidence together with data from clinical studies will increase the level of evidence for the effectiveness and safety of HMPs. Based on this it is conceivable to incorporate HMPs into orthodox therapeutic frameworks.

## Data Availability

'Data was extracted from the pharmaco-epidemiological database PhytoVIS which is owned by the Kooperation Phytopharmaka. Requests concerning the raw data has be addressed to the Kooperation Phytopharmaka Analyzed Data supporting the results can be obtained from the corresponding author. On reasonable request.' Analyzed Data supporting the results can be obtained from the corresponding author. On reasonable request.

## Abbreviations

AOK: Allgemeine Ortskrankenkasse
EMA: European Medicines Agency
HMPs: Herbal Medicinal Products
HMPC: Committee on Herbal Medicinal Products
IMSB: Institute of Medical Statistics and Computational Biology
PROs: Patient-reported outcomes
RWD: Real-world data
uUTI: Uncomplicated Urinary tract infection
